# Computer-aided recording of automatic endoscope washing and disinfection processes as an integral part of medical documentation for quality assurance purposes

**DOI:** 10.1186/1471-230X-10-76

**Published:** 2010-07-08

**Authors:** Bernd Krakamp, Oliver Kirschberg, Andreas Scheding, Dieter Emmerich, Stefanie Klein, Thomas Saers

**Affiliations:** 1 Department of Internal Medicine II, University of Witten/Herdecke, Campus Cologne-Merheim, Ostmerheimerstrasse 200, D-51109 Cologne, Germany; 2 Department of Internal Medicine II, University of Cologne, Campus Cologne-Merheim, Ostmerheimerstrasse 200, D-51109 Cologne, Germany; 3 IT Specialist, Erlangen, Germany

## Abstract

**Background:**

The reprocessing of medical endoscopes is carried out using automatic cleaning and disinfection machines. The documentation and archiving of records of properly conducted reprocessing procedures is the last and increasingly important part of the reprocessing cycle for flexible endoscopes.

**Methods:**

This report describes a new computer program designed to monitor and document the automatic reprocessing of flexible endoscopes and accessories in fully automatic washer-disinfectors; it does not contain nor compensate the manual cleaning step. The program implements national standards for the monitoring of hygiene in flexible endoscopes and the guidelines for the reprocessing of medical products. No FDA approval has been obtained up to now. The advantages of this newly developed computer program are firstly that it simplifies the documentation procedures of medical endoscopes and that it could be used universally with any washer-disinfector and that it is independent of the various interfaces and software products provided by the individual suppliers of washer-disinfectors.

**Results:**

The computer program presented here has been tested on a total of four washer-disinfectors in more than 6000 medical examinations within 9 months.

**Conclusions:**

We present for the first time an electronic documentation system for automated washer-disinfectors for medical devices e.g. flexible endoscopes which can be used on any washer-disinfectors that documents the procedures involved in the automatic cleaning process and can be easily connected to most hospital documentation systems.

## Background

The reprocessing of medical endoscopes is carried out using automatic cleaning and disinfection machines. The complete reprocessing cycle for endoscopes consists of manual preliminary cleaning, fully automatic machine disinfection and cleaning, final visual checking, regular checking of successful hygiene (microbiological surveillance cultures), checking of the hygiene of the endoscope machines (microbiological surveillance cultures), and finally the documentation and archiving of records of properly conducted reprocessing procedures [1]. This complex of reprocessing procedures is set out in the German and European standard DIN/EN/ISO 15883-4 [2,3]. The German standard has been approved by the European standards body, but the most recent version of it, dating from June 2005, has yet to be published and incorporated into European and national legislation.

Several European research groups are involved in establishing a guideline for checking the success of hygiene measures for medical products, and in particular for flexible endoscopes [4-8].

For purposes of quality assurance, all of the required steps set out in the DIN/EN/ISO 15883-4 standard [2,7,8] and in the guidelines for medical products have to be documented, and the records have to be verifiably deposited and stored. This quality assurance procedure can be carried out in handwritten form, using paper print-outs, or in the form of electronic data processing. At present, numerous reporting systems are available that independently link medical systems to the reporting system devices. These include computer-aided reporting systems, printer systems, and also visual systems that have to be transferred in handwritten form.

## Results

The computer program has been successfully applied on a total of four washer-disinfectors in more than 6000 medical examinations within 9 months. The focus of the computer program was to see if it is possible to record automatic endoscopic washing and disinfection processes for medical documentation. The next step is to validate the computer program by statistical examinations. So far, no apparent deficiencies have been reported. The system described here can be reliably used in clinical institutions.

## Discussion and Conclusions

### Concepts of open and flexible documentation

Many different appliance manufacturers are operating alongside each other in the area of automatic washer-disinfectors, supplying comparably efficient products. They are all subject to the regulations applying in the relevant legislation on medical products [4,6]. Technological innovations and the requirements of the relevant standards have given rise to a multiplicity of systems for washer-disinfectors, with various hardware interfaces and the associated proprietary documentation software. Additional systems are likely to be added. The heterogeneous equipment used in endoscopy departments with different washer-disinfectors is not only the result of the fact that there are various manufacturers, but is also due to the time intervals at which new devices are acquired and by replacement purchasing that sometimes involves a change of manufacturer. For the foreseeable future, the various manufacturers cannot be expected to undertake any standardization for the purposes of centralized, consistent and device-independent documentation.

Against this background, we investigated an approach in which all data from the various washer-disinfectors, independently of the hardware interface concerned, can be connected via a device manager.

A common factor in all washer-disinfectors is that documentation data can be output on a standard printer for easy checking by the user. In the simplest case, this printer can be replaced by an electronic connection to the device manager. This includes all of the usual hardware interfaces -- e.g., the parallel printer port, serial port for printing and other data, network interfaces (e.g. TCP/IP and FTP protocol), as well as all of the other typical computer interfaces such as a wireless local area network (WLAN), Bluetooth, etc. For easy checking, all of the washer-disinfectors have to make available to the device manager their processing-relevant data and processing status (e.g., "disinfected", "rinsed"). In addition, the widest possible range of functions, such as the settings and maintenance state of the washer-disinfector, are also supplied to and recorded by the device manager. This allows checking of the maintenance intervals for the washer-disinfectors and analysis of frequent causes of failure in the washer-disinfectors, as well as assessment of frequent error messages affecting the medical products that are being reprocessed. All of the data from the washer-disinfectors (complete recording of all raw data) that are to be output are recorded and stored in a database where they are secure against any revision. These data can be read out at any time. This affects all relevant data such as the time of the start of cleaning, the end of cleaning, documentation of the individual reprocessing steps, the staff member operating the washer-disinfector, the device type with its serial number and cleaning position in the washer-disinfector, and documentation of the washer-disinfector type and its serial number. Status information from the washer-disinfector, such as "cleaned", "disinfected", "leak", "temperature error", etc., are documented and displayed.

Key words defined by the manufacturer of the washer-disinfector, such as "disinfected", are communicated to the device manager from the raw data set and determine whether the medical product or device concerned is released for or withheld from medical use. The medical product is not automatically released for further application unless all safety criteria and procedural steps have been carried out properly and documented. In a graded safety system, the standard user has to register using a bar code before the washer-disinfector is started. The device manager withholds or releases the medical products (e.g., endoscopes) for use, and also the washer-disinfector itself. Both the user and also a specialized statistician can carry out analyses. The administrator of the device manager has to adjust the safety system and check it regularly. This sequential checking system not only meets the requirements of the DIN/EN/ISO 15883-4 standard [2], but in addition makes it possible to carry out statistical and economic analyses. These include frequency of use, frequency of failures of medical products, and the use of the washer-disinfector -- thereby also making it possible to carry out economic testing of the reprocessed medical products and of the washer-disinfector itself. During the reporting process, the medical product is checked once again and released for use.

### Validation of the washer-disinfector and reprocessing procedure and advice-related and batch-related validation

High-quality and reproducible machine reprocessing of medical products by washer-disinfectors is decisively important for the safety of medical procedures and thus also for the patients being examined, and the requirements for this are set out in the guidelines published by the Robert Koch Institute [6]. The same also applies to the relevant hygiene checks (microbiological surveillance cultures) [5,6,9,10]. In relation to medical and medicolegal issues, documentation of the washer-disinfector, including its reprocessing activity, has to be carried out in such a way that it is secure from any later revision.

Monitoring of the reprocessed medical products basically takes place in a batch-related way and by visual checking on the part of the specialist staff. In parallel with this, a record is produced of the cleaning process using process parameters, recorded by a series of sensors and with key words describing the process. The complete reprocessing procedure is documented by the manufacturer of the washer-disinfector using clear key statements in the batch reprocessing record (e.g. "cleaned", "disinfected"). Both negative manual validation and incorrect validation of the cleaning process by the washer-disinfector lead to withholding of the medical product from clinical use. The medical product is only released when there is a positive manual and machine evaluation (Table 1).

**Table 1:** Overview of the functions of the quality assurance program

**Batch documentation**
• Recording of the user and endoscope (type, serial number)
• Documentation and archiving of the entire automatic reprocessing procedure
• Precise assignment of the automatic reprocessing procedure to each endoscope

**Device manager**
• Automatic withholding of the endoscope/medical product if the reprocessing procedure has not been correctly completed
• Automatic withholding of the endoscope/medical product if the set interval (e.g. 5 to 7 days) between reprocessing and use has been exceeded
• Manual withholding of the endoscope/medical product when there are negative hygiene checks or defects
• Automatic recording of the maintenance history and intervals, failures and error messages

**Reporting documentation**
• Linking of the reporting and batch documentation makes additional patient-related documentation of the correctly reprocessed endoscope unnecessary, as it is automatically recorded in the reporting sheet

**Administrator rights**
• Authorization of access to the program is graded
• Correctly reprocessed endoscopes/medical products are accessible to all members of staff for inclusion in reporting
• Manual withholding, revocation of withholding, available analyses and statistical data are controlled in a user-defined and password-secured fashion that is therefore also related to individual staff members


**Analyses available**
• Frequency of use of the washer-disinfector, endoscope, or medical product
• Frequency of errors or failures in the washer-disinfector, endoscope, or medical product
• Maintenance status
• Statistical data

The manufacturers of medical products usually stipulate that devices should be overhauled by the manufacturers on their own premises after a specified number of reprocessing procedures. This applies both to washer-disinfectors and also to endoscopes. For reasons of hygiene, it is sensible to reprocess flexible endoscopes if the endoscopes have not been in use for 5 to 7 days [11-14]. These two fundamental problems -- checking of washer-disinfectors and medical products on the manufacturer's premises, and prolonged storage of the flexible endoscopes -- are defined in the device manager as criteria for withholding the equipment from further clinical use.

**Figure 1:**
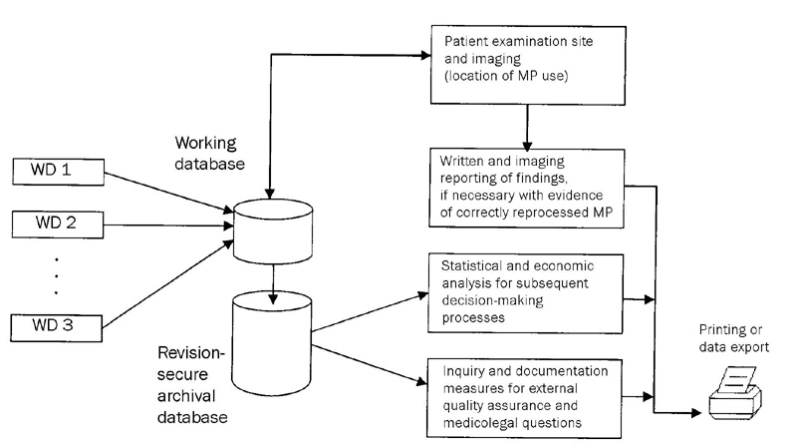
**Diagram showing the structure of the documentation program and information flow**. MP = medical product; WD = washer-disinfector.

Before starting an examination, the computer program requires choosing an endoscope from a pull down menu inside the patient examination site. Only the released medical product (endoscope) is listed and displayed in the patient examination and imaging site. With this step it is secured that only one of those instruments is used for the examination of the patient (Figure 1). This function also allows the physician or staff specialist to get immediately an overview of all directly available endoscopes. Using the device manager's statistical function, the frequency of use, the extent of utilization of a device, and error states in the washer-disinfector and endoscopes can be established for economic analysis.

The usability of a flexible endoscope has to be documented unequivocally and secure from any revision and also has to be automatically monitored. Individual errors have to be electronically checked and avoided in the system presented here. The structure of the newly developed computer-aided quality assurance program is illustrated in Figure 1.

## Methods

In our recently built modern interdisciplinary endoscopy unit, the computer program, syn. reporting system, is now being used to produce all reports in written and imaging form, to communicate them to the hospital information system, and also to print them out as hard copies when needed. It was therefore an obvious additional step to simplify the quality assurance procedures used for the required documentation and to secure the relevant data electronically. This reduces work for the medical staff and also allows the individual working steps involved to be guaranteed by continuous checking.

The computer program, is capable of being used in all medical specialties as a subsystem for medical documentation. It has central data storage with archiving functions and an HL7 interface connection to the hospital information system. In a comprehensive, centralized electronic documentation system, the data from automated washer-disinfectors for medical devices (e.g., flexible endoscopes and accessories) need to be recorded and also correlated with the medical documentation process (e.g., the endoscopic examination). Only properly processed medical products, including endoscopes, may be used in medical procedures.

We now have fully integrated the documentation of automatic cleaning and disinfection into the documentation system using a newly produced computer program. This provides support for the user by automatically documenting all of the steps involved in automatic cleaning and disinfection. The release of medical devices for use is also controlled by the reporting system device manager. In this way it is guaranteed that only proberly disinfected endoscopes are released for examination.

The automatic endoscope washing and disinfection process does not contain nor compensate the manual preliminary cleaning, nor the final visual checking nor the regular checking of successful hygiene. It is one part of the whole process. But the automatic step is independent of individual malpractice and therefore the documentation is also independent.

## Competing interests

The authors declare that they have no competing interests.

## Authors' contributions

BK, DE and TS conceived the design of the computer program and drafted the manuscript. AS, OK and SK participated in its design and coordination and helped to draft the manuscript. All authors read and approved the final manuscript.

## Pre-publication history

The pre-publication history for this paper can be accessed here:

http://www.biomedcentral.com/1471-230X/10/76/prepub
